# Paralytic Shellfish Toxins in the Gastropod *Concholepas concholepas*: Variability, Toxin Profiles and Mechanisms for Toxicity Reduction

**DOI:** 10.3390/md21010044

**Published:** 2023-01-06

**Authors:** Miriam Seguel, Carlos Molinet, Manuel Díaz, Gonzalo Álvarez, Carlos García, Andrés Marín, María Olga Millanao, Patricio A. Díaz

**Affiliations:** 1 Centro Regional de Análisis de Recursos y Medio Ambiente (CERAM), Universidad Austral de Chile, Puerto Montt 5480000, Chile; 2 Instituto de Acuicultura, Programa de Investigación Pesquera, Universidad Austral de Chile, Los Pinos S/N, Puerto Montt 5480000, Chile; 3 Programa Integrativo, Centro Interdisciplinario para la Investigación Acuícola (INCAR), Concepción 4030000, Chile; 4 Facultad de Ciencias del Mar, Departamento de Acuicultura, Universidad Católica del Norte, Larrondo 12181, Coquimbo 1780000, Chile; 5 Centro de Investigación y Desarrollo Tecnológico en Algas (CIDTA), Facultad de Ciencias del Mar, Universidad Católica del Norte, Larrondo 1281, Coquimbo 1780000, Chile; 6 Laboratorio de Toxinas Marinas, Programa de Fisiología y Biofísica, Instituto de Ciencias Biomédicas, Facultad de Medicina, Universidad de Chile, Santiago 8380000, Chile; 7 Centro de Estudios del Desarrollo Regional y Políticas Públicas (CEDER), Universidad de Los Lagos, Osorno 5290000, Chile; 8 Badinotti Group—Chile, Ruta 226, km 9.2, El Tepual, Puerto Montt 5480000, Chile; 9 Centro i~mar & CeBiB, Universidad de Los Lagos, Casilla 557, Puerto Montt 5480000, Chile

**Keywords:** harmful algal blooms, paralytic shellfish toxins, loco, pigment, Chilean Patagonia, MBA, HPLC-FLD PCOX

## Abstract

Harmful algal blooms of toxin-producing microalgae are recurrent in southern Chile. Paralytic shellfish poisoning (PSP) outbreaks pose the main threat to public health and the fishing industry in the Patagonian fjords. This study aims to increase understanding of the individual and spatial variability of PSP toxicity in the foot of *Concholepas concholepas*, Chile’s most valuable commercial benthic invertebrate species, extracted from the Guaitecas Archipelago in Chilean Patagonia. The objective is to determine the effect of pigment removal and freezing during the detoxification process. A total of 150 specimens (≥90 mm length) were collected from this area. The live specimens were transferred to a processing plant, where they were measured and gutted, the foot was divided into two equal parts, and pigment was manually removed from one of these parts. The PSP toxicity of each foot (edible tissue) was determined by mouse bioassay (MBA) and high-performance liquid chromatography with fluorescence detection and postcolumn oxidation (HPLC-FLD PCOX). The individual toxicity per loco, as the species is known locally, varied from <30 to 146 μg STX diHCL eq 100 g^−1^ (CV = 43.83%) and from 5.96 to 216.3 μg STX diHCL eq 100 g^−1^ (CV = 34.63%), using MBA and HPLC, respectively. A generalized linear model showed a negative relation between individual weight and toxicity. The toxicological profile showed a dominance of STX (>95%), neoSTX and GTX2. The removal of pigment produced a reduction in PSP toxicity of up to 90% and could represent a good detoxification tool moving forward. The freezing process in the muscle with pigment did not produce a clear pattern. There is a significant reduction (*p* < 0.05) of PSP toxicity via PCOX but not MBA. Furthermore, the study discusses possible management and commercialization implications of the findings regarding small-scale fisheries.

## 1. Introduction

Paralytic shellfish poisoning (PSP) is a neurotoxic syndrome associated with the consumption of seafood products contaminated with saxitoxin (STX) and its analogues, which can cause a range of symptoms that vary from a slight tingling sensation or numbness around the lips to fatal respiratory paralysis [1]. The toxins involved, known as paralytic shellfish toxins (PST), are biosynthesized by different species of marine dinoflagellates of the genera *Alexandrium* [2], *Gymnodinium* [3], *Pyrodinium* [4] and *Centrodinium* [5]. Currently, more than 57 toxins that are structurally related to STX have been described [6] and these can be divided into six classes: (i) N-sulfocarbamoyl toxins (GTX5, GTX6, C1–C4); (ii) decarbamoyl toxins (dcGTX1–4, dcNeo, dcSTX); (iii) carbamoyl toxins (GTX1–4, neoSTX and STX) [7]; (iv) deoxydecarbamoyl toxins (doSTX, doGTX2–3); (v) hydroxybenzoyl toxins (GC1 to GC6) [8]; and (vi) a series of 11-hydroxy STX analogues referred to as M-toxins [9].

During a bloom of PST-producing dinoflagellates, high concentrations of these compounds can accumulate in bivalve due to their high water filtration rates [10,11,12], thus exceeding the regulatory limit of 80 μg STX eq 100 g^−1^ [13,14]. These can then act as the main transvectors for human intoxication [15]. The toxins contained in dinoflagellate cells are taken up by the bivalves. They are initially stored in the digestive gland and subsequently transferred in part to other organs or non-visceral tissues through the bloodstream [16]. This transfer of toxins can also lead to a variation in the original toxic profile of dinoflagellates through different species-specific biotransformation pathways (enzymatic and non-enzymatic) [17,18] and in certain cases may increase the toxicity of contaminated shellfish [19].

In marine environments, carnivorous gastropods prey on other molluscs, predominantly filter-feeder bivalves, and can therefore accumulate toxins in their tissues [20]. The presence of PST has been reported in different commercial gastropod species worldwide, including *Rapana venosa* [21], *Turbo marmoratus*, *T. argyrostomus* and *Tectus pyramis* in Japan [22], and *Natica vitellus*, *N. tumidus*, *Oliva hirasei*, *O. lignaria* and *O. annulata* in Vietnam [23], among others. In addition, PST have been associated with human intoxication due to the consumption of *Miniaceoliva miniacea*, *Oliva mustelina* and *O. nirasei* in Taiwan [24] as well as fatalities following the consumption of *Nassarius succinctus* and other species of the same genus in China [25]. In South America, PST have been reported in two commercially exploited species on the Atlantic coast of Argentina, which correspond to the carnivorous gastropods *Ademelon beckii* and *Zidona dufresnei* [26,27]. In Chile, PST have historically been reported primarily in two gastropods commercially exploited in Patagonia, *Argobuccinum ranelliformis* and *Concholepas concholepas,* with a maximum toxicity in the digestive gland of 14,057 and 9164 μg STX eq 100 g^−1^, respectively, compared to foot tissue with 31 and 737 μg STX eq 100 g^−1^, respectively [28].

The carnivorous gastropod *C. concholepas* is an endemic species of the Pacific coast of South America, where it is distributed from Lobos Afuera Island, Peru (6°27′ S) to the sub-Antarctic zone (Cape Horn, 56°00′ S) [29]. This gastropod, known in Chile as “loco” or “false abalone”, is a highly valuable economic resource for small-scale Chilean artisanal fishers, with annual landings of approximately 4500 tons, most of which has traditionally been exported in a frozen state to Asian markets [30,31,32,33]. The loco is the flagship species that since the 1990s has driven the implementation and development of the Management and Exploitation Areas of Benthic Resources (AMERB, by its original Spanish acronym) system, based on co-management and the territorial use rights for fisheries (TURF) principles [34]. Nowadays, the loco can be exclusively extracted from AMERB by licenced fishing organizations in accordance with individual management plans and subject to State control [35].

Along the Chilean coast, the majority of the landing areas correspond to the Coquimbo Region (901 tons/year) and the Los Lagos Region (1041 tons/year) [36]. Different levels of PST have been recorded in both areas. In Coquimbo, PST toxicity has never surpassed the regulatory limit and is not expected to have a relevant impact on the culture and exploitation of this species [37]. However, in Patagonia, higher toxicities have been reported in *C. concholepas* populations, mainly in the Aysen and Magallanes Regions, where prolonged bans for harvesting have been implemented. As a consequence, landings of benthic resources in these regions have dramatically decreased in recent years and decades [28,38,39]. Indeed, 2016 saw the worst event of PST toxicity in Chilean history in terms of geographical extension, fisheries impacted and aquaculture mollusc-affected species harmed [40]. The event had severely negative socioeconomic consequences across local economies [41,42,43]. During the outbreak, *C. concholepas* populations remained contaminated for more than 18 months after the bloom, which suggests that this particular gastropod is a slow detoxifying organism [20,44]. Under such conditions of PST-toxified loco, and depending on specific toxicity levels (i.e., with regard to regulatory thresholds), fisheries and/or fishing organizations may decide when to harvest and whom to sell (i.e., intermediaries or directly to processing plants). It should be noted that only detoxified loco can be frozen in order to comply with Asian food safety standards. However, this practice can delay harvests and earnings until the following season. Loco with toxicity levels close to the threshold (i.e., 80 µg STX eq 100 g^−1^ meat) can be canned for retail, although this reduces the sales price and therefore negatively affects fishers’ earnings. Until recently, fishers have had limited options in terms of reducing toxicity levels, negotiating better prices and sustaining their productivity during and after harmful algal bloom (HAB) events. Therefore, improved management measures are needed with which to mitigate the negative consequences of PST episodes in fisheries and the commercialization of this particular gastropod.

Certain studies have focused on the interindividual and anatomical distribution of PST in gastropods in order to develop specific treatments with which to reduce or eliminate toxins from edible tissues. For example, in the carnivorous gastropod *Zidona dufresnei*, an important commercial species on the South American Atlantic coast, high toxicities have been reported in its visceral mass (2247 µg eq STX 100 g^−1^) and foot (337 µg eq STX 100 g^−1^) [27]. To decrease the toxicity of the final product, selective evisceration of visceral mass and thermal treatments were evaluated during the canning process and, as a result, a safe product for consumption has been obtained. However, recommendations are that the toxicity of animals collected in their natural environment be close to the regulatory limit in the muscle and four times higher in visceral mass [45,46].

Another species that is commercially important is the abalone *Haliotis tuberculata*. This species can accumulate PST with toxicity levels above the regulatory limit in its edible tissues. The anatomical distribution of toxicity, in increasing order, has been reported in the foot (27 µg eq STX 100 g^−1^), intestine (29 µg eq STX 100 g^−1^) and epithelium of the foot (10,500 µg eq STX 100 g^−1^). Therefore, the authors of the research behind these findings suggest that the removal of the foot epithelium would enable its exploitation off the Galician coast and its marketing across Europe [47,48,49].

To date, certain studies have been compiled to determine the anatomical distribution of toxicity in *C. concholepas*. For example, Compagnon, et al. [28] reported that the toxicity in the visceral mass (non-edible tissues) and foot (edible tissues) of individuals from the Aysen Region were 9164 and 737 μg STX eq 100 g^−1^, respectively. Conversely, Oyaneder-Terrazas, et al. [50] found that individuals from the same geographical area had higher toxicity in the foot (600 µg eq STX 100 g^−1^) than in visceral mass (450 µg eq STX 100 g^−1^). Consequently, further research is needed to clarify the anatomical distribution of PST in this species in order to facilitate the development of specific treatments to reduce or eliminate toxins from edible tissues.

With that in mind, the objectives of the present study were as follows: (i) to analyse the individual variability of PST and toxin profiles in edible tissues of *C. concholepas*; and (ii) to evaluate mechanical (removal of pigment through brushing) and physical (freezing) processes to reduce the toxicity of PST in edible tissue of *C. concholepas*, as well as potential changes in its toxin profile. The findings of this work may be useful in reducing the socioeconomic impacts of recurrent PSP events in southern Chile and other parts of the world.

## 2. Results

### 2.1. Size Structure of C. concholepas

**Figure 1:**
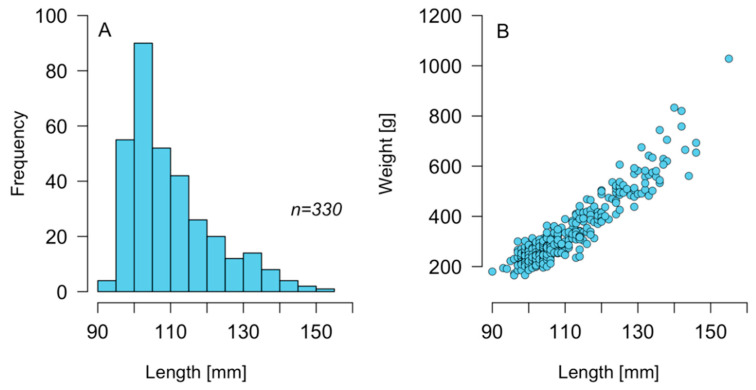
(**A**) Size histogram and (**B**) length-weight relation recorded in loco *Concholepas concholepas* extracted from the Guaitecas Archipelago, November 2018.

Individual specimens of *C. concholepas* extracted between 6 and 7 November 2018 around the Guaitecas Archipelago presented a range of size and weight between 90–155 mm (Figure 1A) and 165–1028 g (Figure 1B), respectively. The average size of the population sampled was 110 mm, while the average weight was 335 g. A large part of the sampled population (88.5%) had a size equal to or greater than the minimum legal size for extraction (100 mm).

### 2.2. Toxicity and Toxin Profile in Potential Prey

**Figure 2:**
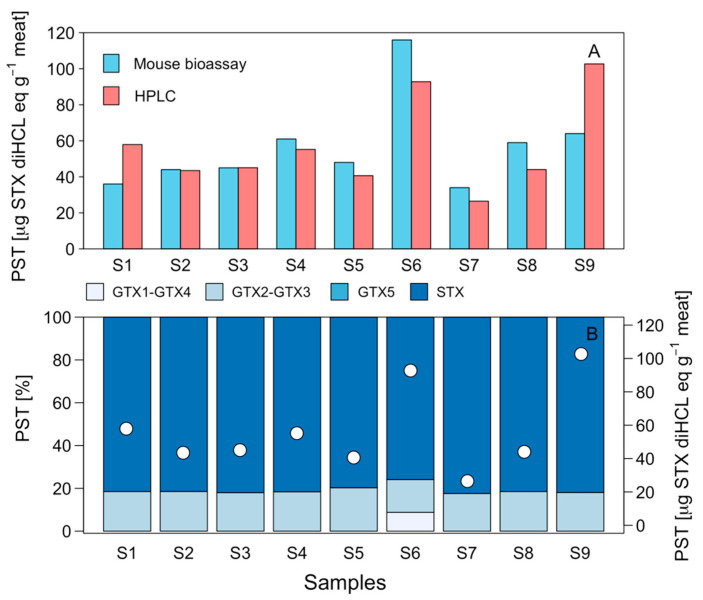
(**A**) Paralytic shellfish poisoning (PSP) toxicity (μg STX eq 100 g^−1^) recorded through the mouse bioassay (light blue bars) and HPLC-FLD PCOX (pink bars) techniques, and (**B**) toxin profile (%; blue scale bars) and toxicity (μg STX diHCL eq 100 g^−1^; full white circles) in nine samples of mussel *Mytilus chilensis* extracted from the Guaitecas Archipelago, November 2018.

Samples of mussels *Mytilus chilensis*, a potential prey of *C. concholepas*, evaluated by means of mouse bioassay (MBA) showed toxicities to range from 34 to 116 μg STX eq 100 g^−1^ (Figure 2). The results obtained through the high-performance liquid chromatography with fluorescence detection and postcolumn oxidation (HPLC-FLD PCOX) technique show similar results, with toxicities that varied between 26.5 and 102.7 μg STX diHCL eq 100 g^−1^ (Figure 2A). The toxin profile was dominated (>80%) by STX, followed by GTX2-GTX3 (Figure 2B). The presence of GTX1-GTX4 was also detected, although in a low percentage (8.8%) and in only one sample. Nevertheless, a clam (*Ameghinomya antiqua*) sample that was obtained from this same sector had a toxicological profile that was dominated by GTX1-GTX4 (~78% and a total toxicity of 43.3 μg STX diHCL eq 100 g^−1^ (data not shown).

### 2.3. Interindividual Toxicity in Edible Tissues

**Figure 3:**
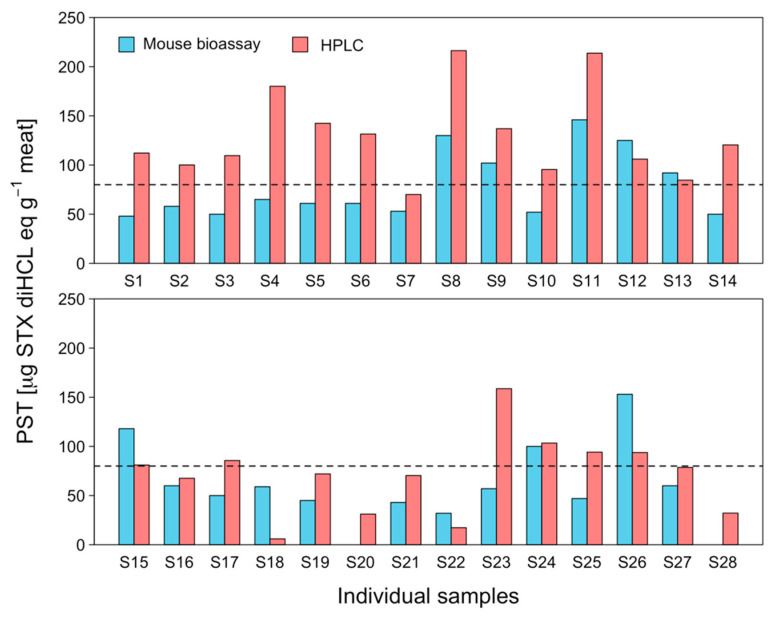
Interindividual toxicity of *C. concholepas*, obtained through the mouse bioassay (light blue bars) and HPLC-FLD PCOX (pink bars) techniques, extracted from the Guaitecas Archipelago, November 2018.

**Figure 4:**
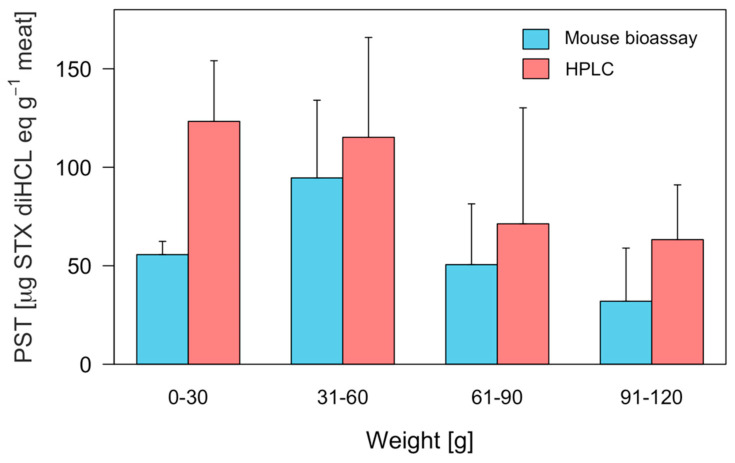
PSP toxicity of different weight groups of *C. concholepas* edible tissues (foot with pigment) obtained through the mouse bioassay and HPLC techniques, extracted from the Guaitecas Archipelago, November 2018.

The determination of individual PST toxicity in edible tissues (foot) using the MBA and HPLC techniques showed a large degree of variability (Figure 3). In the case of MBA, toxin toxicity ranged from below the detection limit (<30 μg STX eq 100 g^−1^) to 146 μg STX eq 100 g^−1^, with a coefficient of variation (CV) of 43.83%. On the other hand, the HPLC technique produced scores ranging from between 5.96 and 216.3 μg STX diHCL eq 100 g^−1^ with a CV of 34.63% (Figure 3). Thus, 21 out of 28 individuals registered higher toxicity values using the PCOX technique (Figure 3). In addition, the number of individuals that registered toxicity higher than the permissible limit for human consumption (80 μg STX eq 100 g^−1^) by means of the MBA and PCOX techniques was 8 and 19, respectively (Figure 3). When comparing the toxicity results obtained at the individual level with the two techniques used, i.e., those of MBA and PCOX, it was noted that differences were more pronounced among smaller individuals (Figure 4). An ANOVA evidenced the significant effect of techniques used (MBA and PCOX) and weight on toxicities (*p* < 0.05).

### 2.4. Effect of Foot Pigment Remotion Process on PST Detoxification

**Figure 5:**
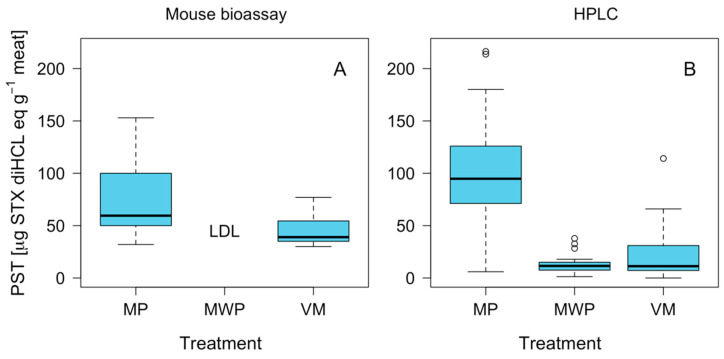
Boxplot of PSP toxicity in loco *C. concholepas,* recorded through the mouse bioassay (**A**) and HPLC-FLD PCOX (**B**) techniques using muscles with pigment (MP), muscles without pigment (MWP) and visceral mass (VM), extracted from the Guaitecas Archipelago, November 2018. LDL: below the detection limit. The circles represent the extreme values (outliers).

Analyses to determine differences in toxicity in relation to the foot with pigment (FP) and foot without pigment (FWP) variables showed a significant decrease (*p* < 0.01) when the pigment was removed by brushing (Figure 5A). This trend was repeated across both the MBA and PCOX techniques. In the case of the MBA technique, toxicity levels of pigmented foot (146 μg eq 100 g^−1^) were reduced to levels below the detection limit (<30 μg STX eq 100 g^−1^) following pigment removal by brushing, representing a toxicity reduction of at least 80% (*p* < 0.01). This reduction is particularly significant (*p* < 0.01) when comparing toxicity samples through the PCOX technique, in which the reduction levels reached up to 92% (Figure 5B).

**Figure 6:**
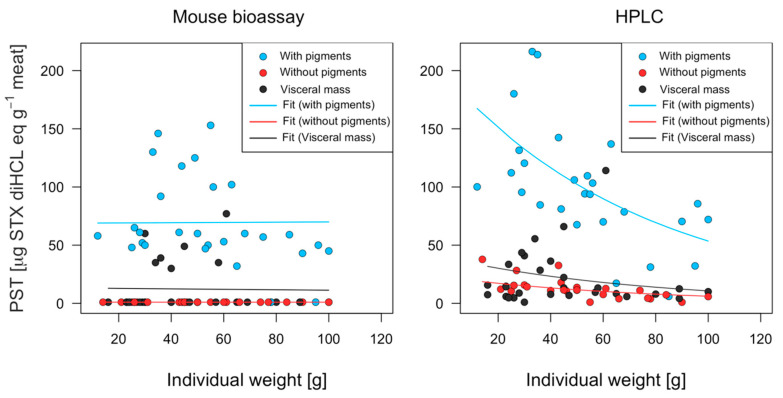
Observed and fitted toxicity of *C. concholepas*, obtained through the mouse bioassay and HPLC-FLD PCOX techniques, extracted from the Guaitecas Archipelago, November 2018.

A generalized linear model (GLM) was used to show a negative relationship between individual weight and toxicity estimated by the PCOX technique (Figure 6). The discrepancies in toxicity recorded by the two techniques (MBA and PCOX) can be explained by the greater accuracy of PCOX. While a correlation was observed between individual weight and toxicity in the results of the HPLC analysis, this effect was not detected via the bioassay analysis (Table 1 and Figure 6). Thus, the most informative model with which to explain toxicity variability from the MBA technique was a generalized Gamma model, while, for its PCOX counterpart, it was a generalized negative binomial (Table 1).

The deviance analysis of negative binomial model applied to study toxicity from the bioassay (Table 2) and HPLC-FLD PCOX (Table 3) showed a clear effect of treatment (*p* < 0.05).

**Figure 7:**
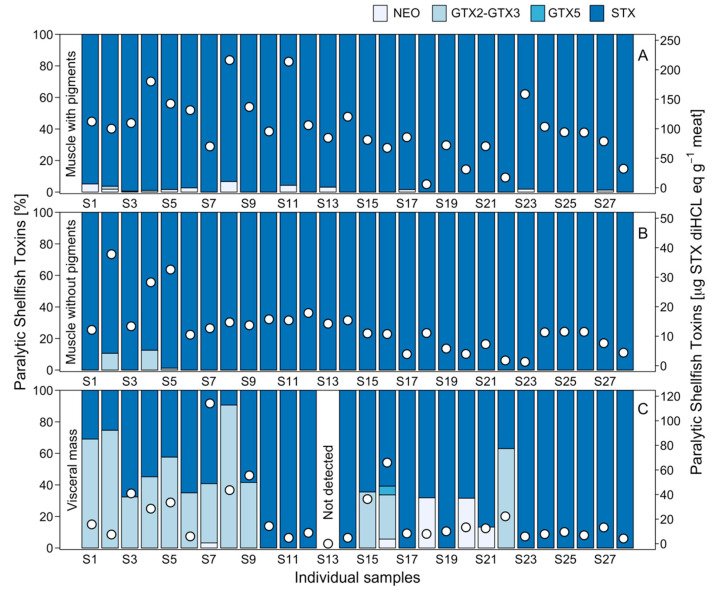
PSP toxicity (white circles) and toxin profile (blue bars) recorded in individual samples of *C. concholepas* through the HPLC-FLD PCOX technique using pigmented muscle (**A**), unpigmented muscle (**B**) and visceral mass (**C**) extracted from the Guaitecas Archipelago, November 2018.

When analysing the toxin profiles of FP (muscle) and FWP, as well as the viscera of different individuals, a number of interesting observations were made (Figure 7). Although the elimination of the pigment generated a significant reduction in toxicity, no change in the toxin profile was observed. Thus, the toxin profile of the FP and FWP was dominated by STX. A smaller proportion (<5%) of neoSTX was detected in certain pigmented foot samples and GTX2 (<10%) in unpigmented foot samples. Nevertheless, in the case of visceral mass, although STX was highly dominant in the majority of the samples, some showed a higher presence of GTX2-GTX3, representing more than 50% in three individuals (Figure 7; Figure A3).

### 2.5. Effect of Fresh-Frozen Process on PST Detoxification

**Figure 8:**
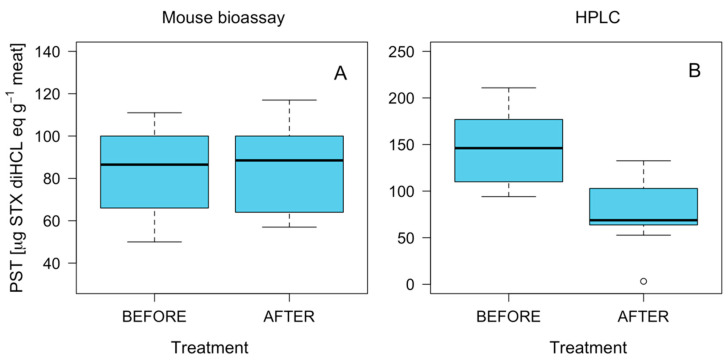
Boxplot of PSP toxicity in *C. concholepas* recorded through the mouse bioassay (**A**) and HPLC-FLD PCOX (**B**) techniques prior to (initial) and after (final) the freezing process in individuals extracted from the Guaitecas Archipelago, November 2018.

**Figure 9:**
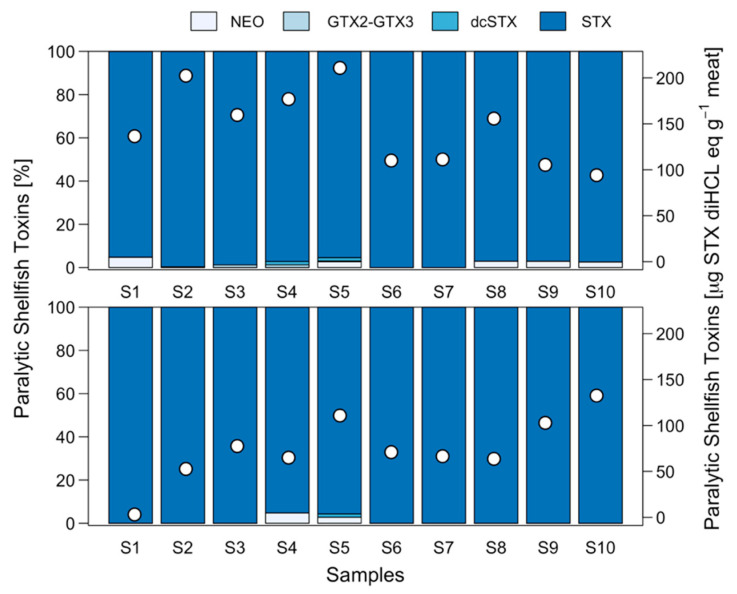
PSP toxicity (white circles) and toxin profile (blue bars) recorded in individual samples of *C. concholepas* through the HPLC-FLD PCOX technique using pigmented muscle prior to (upper panel) and after (lower panel) the freezing process, extracted from the Guaitecas Archipelago, November 2018.

Results on toxicity levels in pigmented muscle prior to and after the freezing process showed no clear pattern. In the case of the MBA technique, no significant differences were observed in toxicity owing to the effect of the freezing process (Figure 8). However, the HPLC-FLD PCOX results showed a significant reduction in toxicity (*p* < 0.05). The toxin profile was stable in the samples prior to and after the freezing process, showing no significant differences. The toxin profile was dominated by STX, although traces of neoSTX, dcSTX and GTX2-GTX3 were also detected (Figure 9).

## 3. Discussion

### 3.1. Toxin Distribution and Mechanisms for Toxicity Reduction

The gastropod *C. concholepas* is a carnivorous gastropod endemic to the Pacific coasts of Peru and Chile. It is traded primarily to Asian markets (Taiwan, Hong Kong and Japan) as a substitute for abalone (genus *Haliotis*). The muscle is marketed and exported under the raw-frozen (55%) and canned (44%) processing lines [51]. In the case of the raw-frozen line, the muscle must include the green–blackish pigment, which surrounds the entire tissue, since it is this pigment that gives the product a fresh appearance. In addition, the foot is covered by the mucosa, which fulfils an important fusion function for attachment and mobilization purposes [52].

The general mechanism by which carnivorous gastropods accumulate toxins has been described by Shumway [20]. In the case of *C. concholepas*, the feeding mechanism includes both boring into and attacking live prey by manipulating it with the foot [53]. Therefore, after the ingestion of prey toxins and its temporary accumulation in the digestive gland, toxins are mobilized by the circulatory system to foot tissue, and in particular in the pigment. This explains the observed toxin concentration in the foot as well as in the digestive gland recorded in this study and those undertaken by Compagnon, et al. [28] and Molinet, et al. [54]. It appears that the mucus secreted by the loco, which also lubricates the foot, plays a key role in the distribution of toxins within the foot and probably also play a role in the retained of PST toxins for a long period of time [28].

To date, different mechanisms of detoxification of paralytic toxins in bivalve molluscs have been described as reducing toxicity to a value below the permissible limit for human consumption, including evisceration, freezing and high temperature processes [55,56,57,58]. In certain products, such as the scallop *Pecten maximus*, the combination of evisceration and thermal processing produces a decrease of toxicity from 300 μg to values below the detection limit [56].

The detoxification mechanisms employed by carnivorous and herbivorous gastropods are also based on the elimination of the most toxic tissues or thermal processes in the final product manufacturing. For example, it has been determined that the concentration of toxins in the carnivorous snail *Zidona dufresnei* has the following descending pattern: viscera > epithelium > mucous secretion > muscle [27]. In the abalone *Haliotis tuberculata,* the toxicity in the epithelium is 300 times higher than in the muscle and stomach contents [47]. Therefore, the removal of this particular body part could serve as a detoxification mechanism, particularly in the canning line. The results of this study also show that the removal of the pigment produced a decrease of approximately 90% of the original toxicity as has been previously demonstrated by Dowsett, et al. [59] in the abalone *H. laevigata* and by Seger et al. [60] in *H. rubra rubra*.

The brushing procedure applied to the foot of the loco in this study resulted in a toxicity reduction of approximately 90%, from around 200 μg STX diHCL eq 100 g^−1^ to < 50 μg STX diHCL eq 100 g^−1^ using the HPLC-FLD PCOX technique and at values not detected by means of the MBA method. This suggests that the highest toxicity in the foot muscle is associated with the pigment. Therefore, the removal of this particular body part could also be used as a detoxification procedure, once more in the canning line. Furthermore, the negative relationship between the individual weight of the loco and its foot-toxin concentration that resulted from the PCOX analysis (during the detoxification stage observed) may be explained by the following: (i) the allometric relationship between the foot-exposed surface (zone with pigments) versus weight or (ii) the possible matrix effects in samples from the smallest individuals. This latter point is an area that requires further study.

The fresh-frozen process line is the most important one for this product from an economic perspective. As a result, evaluations of the effect of freezing on the concentration and toxicological profile are necessary. Freezing is a process rarely used in the canning industry since the decrease of toxins via this method is lower and occurs via their elimination through the thawing water [56]. In this study, the freezing process does not show a significant effect on decreasing toxicity when analysed using the MBA technique, but it does produce interesting findings by means of the PCOX technique on individuals extracted from the Guaitecas Archipelago. It should be noted that in the raw frozen process line, following a few hours of freezing, a glaze is applied with fresh water to form a protective barrier which helps to avoid product dehydration. In addition, freezing does not change the toxicological profile of the product and saxitoxin remains dominant therein (data not shown). This is different in high-temperature thermal processes, whereby there is transfer of toxins from the meat to the covering liquid, and, consequently, a transformation in the toxicological profile occurs [55,56,57,58].

### 3.2. Biological and Chromatographic Analyses: Potential Implications

Toxicity analyses in samples of the edible tissues of *C. concholepas* revealed differences between the MBA and PCOX techniques, with an overestimation of 38% toxicity for the chromatographic technique compared to the bioassay, which in certain cases surpassed the regulatory limit. In contrast, such differences were not as clear in the samples of mussels (*Mytilus chilensis*) obtained from the same study area, which may constitute potential source of prey for *C. concholepas*.

Comparison studies between the PCOX and the MBA technique have shown trends that differ on the basis of the type of shellfish analysed [61,62]. The available interlaboratory comparison with Chilean samples showed that MBA techniques overestimate toxicity compared to the PCOX technique, particularly in bivalve molluscs, with differences of one order of magnitude in clams (*Gari* solida) and two or three orders of magnitude in mussels (*Mytilus chilensis*) and oysters (*Magallana gigas*) and with similar results observed in scallops (*Argopecten purpuratus*) [63].

At present, there is no information about the differences in PST toxicity between techniques used in Chilean gastropods. Consequently, this study represents the first comparison of this group of molluscs. In this context, it is possible that the toxicity overestimation of the PCOX technique could be explained by matrix effects of edible tissues (foot) in *C. concholepas*, which may suggest that sample pre-treatment (i.e., solid phase extraction (SPE) purification or protein precipitation) would be necessary to reduce or eliminate matrix interference. Studies which demonstrate overestimation of PST toxicity by PCOX methodology are scarce. Nevertheless, Hignutt [62] reported an at least threefold increase of toxicity in viscera of the Dungeness crab (*Metacarcinus magister*) in Alaska, suggesting that the PCOX method is not currently suitable for analysis of PSTs due to the presence of an unknown matrix interference unique to this species.

An interesting aspect observed in this study was the relationship between high toxicity and animal size. In this regard, the smaller individuals were more toxic than the larger ones, particularly in the samples analysed by means of the PCOX technique. A similar tendency was previously observed in the abalone *Haliotis tuberculate* in the research conducted by Bravo, et al. [47], who described that, through the deployment of the PCOX technique, the smallest individuals had more toxicity than the largest specimens, although this particular trend was not appreciable with regard to the use of the MBA technique.

Considering this background, there are certain important implications with regards to the current monitoring systems for marine toxins in Chile. Presently, PST toxicity is evaluated using MBA. However, in order to comply with safety requirements and commercial agreements, the implementation of chromatographic techniques may be necessary. Therefore, interlaboratory studies are needed to establish correlations between analytical methodologies and MBA in gastropod samples. Moreover, further research is required to improve methodologies that eliminate the matrix effect in *C. concholepas* samples to support the proposed methodology to reduce toxicity in the edible tissues of *C. concholepas*.

### 3.3. Implications for Management and Commercialization

The results of this study have important implications for the small-scale fishers and fishing communities that depend on the highly valued Chilean loco in the face of PSP algal blooms. Fisher organizations that co-manage loco exploitation under the AMERB system commonly rely on the harvesting season to extract and commercialize their annual produce (i.e., on the basis of the “total allowable catch” [64]). However, their plans frequently have to be postponed due to species contamination. As a slow detoxifying species, the loco can retain PSP toxins for long periods of time (up to 18 months) following the cessation of HAB concentrations in coastal waters [20]. With toxicity levels above 80 μg STX eq 100 g, harvests and sales are prohibited and the revenue of fishers is delayed until sanitary conditions recuperate. The findings of this study have shown that a relatively simple processing technique, i.e., a 3–4 min brushing to remove the dark pigment, can significantly reduce loco toxins by up to 80–90%. This treatment can potentially ensure that otherwise contaminated extractions fall below the regulatory and/or bio-essay detection limits. Loco brushing represents a concrete and reliable response to PSP events and one that can substantially reduce the negative socioeconomic impacts of HABs on local economies.

However, the removal of the dark pigment from the loco will not resolve all the problems facing fishers and traders and may indeed pose broader commercial challenges on the fishery. For example, the pigment is considered a desirable attribute and a freshness indicator of frozen loco by consumers in East Asian markets. Its removal for sanitary purposes would imply a different final product, namely canned or conserved loco [65]. In turn, canned loco may imply lower sales price and earnings, require greater local and national processing capacity and could result in the need to explore new markets as the global demand falls.

The following two paragraphs illustrate the implications of the findings of this study at the practical and real-life level. The examples are based on responses from field interviews and relate to two contrasting experiences following the 2016 bloom in the Caleta Mississippi and Caleta Estaquilla communities, in the Los Rios and Los Lagos Regions, respectively.

The first example dates from August 2017, when fishers from Caleta Mississippi (CM) received the first post-bloom sanitary permission to extract 6000 units of loco from their AMERB, since recent bioassays had returned favourable results. Prior to harvesting the catch, leaders of the fisher groups had agreed a price of USD 1.2 per unit with a processing plant at the landing site, for freezing and export purposes (MV, male and representative; Pers. Comm.). However, following the harvest and during the transaction, the fishery authority informed that latest bioassays for the area had reported toxins slightly above the permitted limit (81 μg STX eq 100 g), which therefore impeded the commercialization of frozen locos. Since the plant had neither the capacity nor interest to export canned loco, the deal could not be completed. Other processing plants in Chile with canning facilities were overstocked and also disinterested. Consequently, the loco catch from CM was stored out of the water in two boats with no final destination, and in such circumstances, decomposition begins after a maximum of five days. Although it is unconfirmed whether their produce was returned to the sea or illegally sold in the informal local/regional markets [66], the costs of a stranded harvest, lost profits and the resultant socioeconomic impacts on the families of the fourteen fishers involved are clear (MV, male and representative). In addition, the informal sale of PSP-contaminated loco is likely to lead to grave health consequences. The existence of local brushing knowledge and a greater processing capacity would have made a difference for the CM fishing community and shellfish consumers in the face of such adverse circumstances.

The second example dates from May 2016, when, in the middle of the large-scale PSP bloom and crisis mentioned in Section 1, fishers from Caleta Estaquilla (CE) had agreed to sell 90,000 loco units to a processing plant for freezing and export purposes (ZB, female and representative). Once the produce had been placed in cold storage but prior to payment being made to the fishers according to the agreed price (USD 1.25 per unit), the processing company detected higher proportions of toxified individuals than was permitted by external markets. This would dramatically reduce the value of the locos and possibly impede their commercialization altogether. CE fishers claimed their locos back from the plant, although more than 20% of the produce was retained in order to cover storage costs, according to the processing company. Finding an alternative means of commercialization was not straightforward given the limited installed capacity in the local area, the strict sanitary regulations and the economic risk posed by unexplored markets. After six months of trying, CE fishers in conjunction with a medium-sized enterprise, obtained the required legal and sanitary permission to process and commercialize jarred loco in the internal market. The key was that they had learned about the toxin-reduction effect of brushing from a Chilean professional working in the seafood industry in China. The process of loco brushing, washing and freezing led to the PSP level of the loco catch in question to fall below bioassay detection limits and thus allowed the organization to obtain a higher price for their produce (USD 1.6 per unit) than initially offered by the export firm. Jarred locos from CE were successfully sold on the regional and national scale, and even commercialized by a large and reputable retailer (the Jumbo supermarket chain). Despite PSP toxins and a range of trading, regulatory and processing barriers, the 75 fishers and their families took full advantage of the brushing technique and innovated a new product in a relatively new market. Moreover, they reported highly positive socioeconomic results (ZB, female and representative).

Moving forward, further efforts will be necessary to develop shellfish processing capacity at the local and regional levels. In addition, new marketing strategies are needed in order to expand and consolidate the demand for canned loco in Chile and other potential markets.

## 4. Material and Methods

### 4.1. Study Area

**Figure 10:**
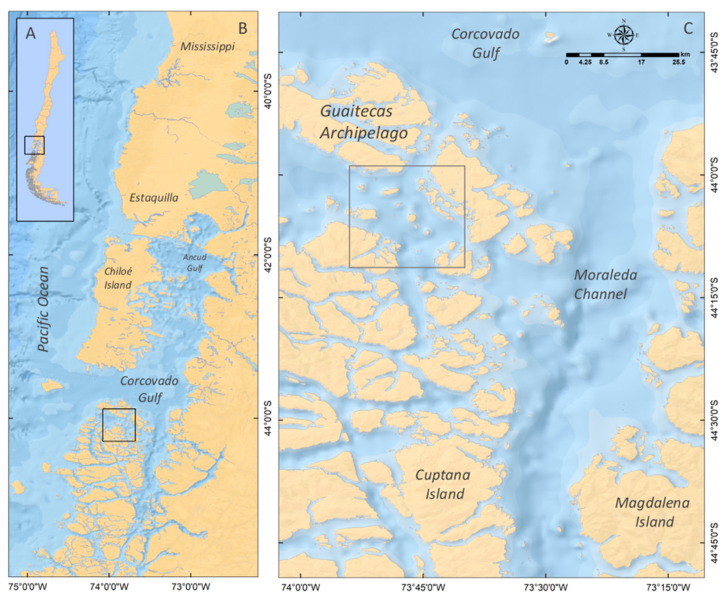
Map showing: (**A**) Chile. (**B**) Chilean Inland Sea, including location of the study area (black rectangle). (**C**) Guaitecas Archipelago where loco individuals were extracted.

The study area for this research is located in the Guaitecas Archipelago (44° S–44°15′ S; Figure 10). This area forms part of the channel system of north-western Patagonia, which is characterized by an abrupt bathymetry and a complex coastal morphology that are both strongly shaped by oceanic water [67].

The loco fishery, and benthic fisheries in general, are of minor socioeconomic significance for coastal fishing communities in the Aysen Region, precisely due to the persistence of high levels of PST toxins [42]. Therefore, Patagonian fjords represent an interesting setting in which to enhance understanding of detoxification processes and toxicity reduction mechanisms regarding the loco compared to other regions with more intensive AMERB operations.

### 4.2. Samples Collection

Between 6 and 7 November 2018, *C. concholepas* samples (size >90 mm) were collected from the Guaitecas Archipelago (Figure 10C). Two experiments to evaluate toxicity reduction and potential changes in the toxin profile were carried out as follows: (i) to analyse the individual variability of PST and toxin profiles of *C. concholepas*; and (ii) to evaluate mechanical (removal of pigment through brushing) and physical (freezing) processes to reduce the toxicity of PST, as well as potential changes in the toxin profile, in edible tissues of *C. concholepas*.

The sampling site was georeferenced with a global positioning system. Simultaneous to the extraction of loco samples, samples of a potential prey (the mussels *Mytilus chilensis*) were collected and also subjected to mouse bioassay and HPLC analysis in accordance with the methodology described in Section 4.3 (MBA) and 4.4 (HPLC). This allowed for comparisons to be made between the toxicological profiles found in the *C. concholepas* and its prey.

#### 4.2.1. Experiment 1: Individual Variability of Toxicity and Effect of Pigment Extraction

**Figure A1:**
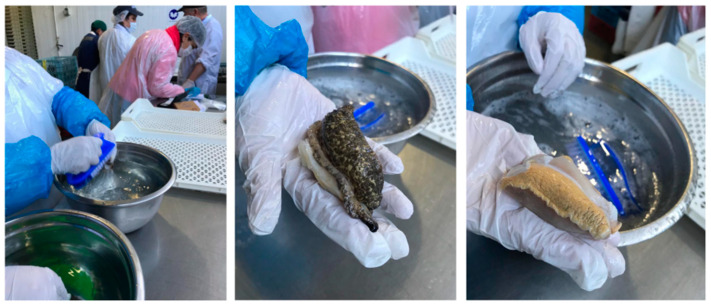
Brushing process to remove pigment from 50% of foot.

A total of 28 individuals of *C. concholepas* were collected to determine individual variability toxicity of edible (foot) and non-edible tissues (visceral mass) by means of MBA and HPLC analysis (see Section 4.3 and Section 4.4). The toxin profile was determined using the latter method. Each individual was weighed and measured, and then the individuals were carefully dissected into the foot (edible tissue), and the viscera (non-edible tissues) registered the weight of each sample. The foot of each sample was divided into two halves and one half was carefully labelled and stored without additional manipulation. The other half of the foot underwent pigment removal using a brush for approximately 3–4 min (Figure A1). This action was performed for all 28 samples. Subsequently, the samples were sent to the laboratory for analysis purposes. Toxicity of visceral mass, foot with pigment and foot without pigment were evaluated by means of MBA and determined through HPLC.

#### 4.2.2. Experiment 2: Effect of the Freezing Process

In contrast to the tests to determine individual variability of edible tissue (foot) (Experiment 1), in this experiment the samples (20 samples of 4 individuals each; n = 80 individuals) were transferred to the processing line. This was done to avoid mixing individual locos and to ensure their traceability.

**Figure A2:**
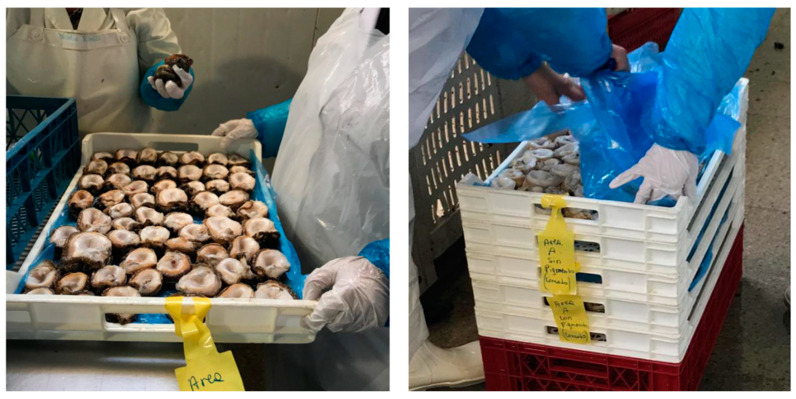
Fresh-frozen process performed on locos with pigment at Pacific Farmer processing plant (Calbuco, Chile), November 2018.

**Figure A3:**
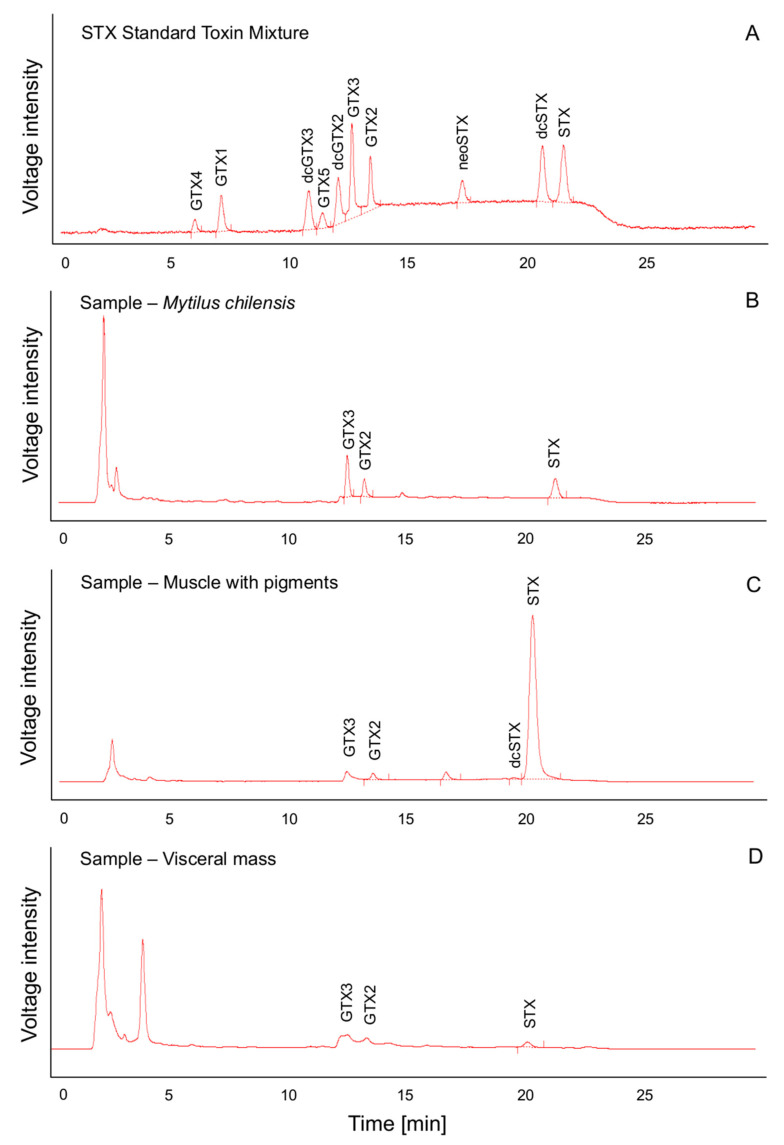
HPLC-FLD PCOX chromatograms of the (**A**) STX standard toxin mixture. (**B**) Sample of the mussels *Mytilus chilensis*. (**C**) Sample of the muscle with pigment of *Concholepas concholepas*. (**D**) Sample of the visceral mass of *C. concholepas*.

To evaluate the fresh-frozen process, tests were carried out to assess the effect of freezing on the toxicity of *C. concholepas*. The tests were carried out in the same manner as a normal fresh-frozen process in the plant selected for this study. Therefore, each individual was shucked and eviscerated. Subsequently, having undergone no further form of manipulation the foot was placed in plastic trays, which were carefully labelled according to the number of individual (Figure A2). The experiment was carried out analysing 10 replicates (4 individuals each) prior to and after freezing process.

Prior to freezing, foot samples (edible tissue; whole individual) were taken from each of four individuals for toxin analysis. Each group of trays that had been subjected to the different treatments was marked and placed in a cold chamber for five hours to reach thermal equilibrium at −18 °C, the core temperature of the product. Once this time had elapsed, the individuals were taken to the packing sector, where samples were taken for each treatment (after freezing). Next, the five samples from each of the four individuals were sent to the laboratory. The remaining individuals were glazed, a procedure that consisted of submerging each one in fresh water to form a protective barrier that helps to prevent against product dehydration. The samples were then packed in plastic bags and boxes.

### 4.3. Toxicity Evaluation by Biological Method Analysis

The toxicity of *C. concholepas* was evaluated by MBA following the Association of Official Analytical Chemists (AOAC) method 959.08 [68]. For toxin extraction, 100 g of homogenized raw tissues was mixed with 100 mL of HCl (0.1 N) solution using a blender, and then boiled for 5 min. The sample was cooled at room temperature for 10 min prior to the pH being corrected to between 2 and 4. At the end of the procedure, the resulting extract was transferred to a 200 mL volumetric flask and filled to the limit with HCl (0.003 N). Aliquots (1 mL) of the final extract were intraperitoneally injected into three Swiss mice weighing 19–21 g, following the official AOAC method 959.08, and their times of death were recorded. If any mouse died in less than 5 min the test was performed again using diluted samples until the mouse died within 5 to 7 min. The toxicity was calculated and expressed as µg STX eq 100 g^−1^ sample using Sommer’s Table. The detection limit of the bioassay was 30 µg STX eq 100 g^−1^.

### 4.4. Toxicity and Toxin Profile Determination by Chromatographic Analyses

Each tissue was extracted following the official AOAC method 2011.02 [69], albeit with slight modifications. Extraction was performed with HCl (0.1 N) (1:1, *w/v*) using an Ultra-Turrax T25 dispersing system (IKA^®^Werke GmbH & Co. KG, Staufen, Germany) at 11,000 rpm for 3 min. After each extraction, the pH of the resulting emulsion was adjusted to between 2 and 4, boiled at 100°C for 5 min, centrifuged at 5000× *g* for 20 min (Centurion K2015R, Centurion Scientific Ltd., Stoughton, West Sussex, UK) and its pH adjusted again to between 2 and 4. To deproteinate the samples, a 1 mL aliquot was mixed with 50 μL of 30% trichloroacetic acid (TCA), vortex-mixed and then centrifuged at 9000× *g* for 5 min. To neutralize the solution, 70 μL of NaOH (1 M) were added and then vortexed and centrifuged at 9000× *g* for 5 min. One 500-μL aliquot was filtered through a 0.2 μm Clarinert nylon syringe filter (13 mm in diameter) (Agela technologies, Torrence, CA, USA) and stored in an autosampler vial. A second 500-μL aliquot was hydrolyzed to transform the sulfocarbamate toxins (if present) to the corresponding carbamate toxins (HCl 0.4 N, 100 °C, 15 min), filtered through a 0.2 μm nylon filter and stored in an autosampler vial. For certain analyses, extracts were diluted fivefold or tenfold with HCl 0.1 N to ensure the correct quantification of the most abundant toxins in the samples. The most important PST toxins were quantified following the official AOAC method 2011.02 by HPLC using a Hitachi LaChrom Elite HPLC system equipped with a Hitachi FL detector L2485 (Hitachi High Technologies America Inc., Chatsworth, CA, USA) and a post-column reaction system composed of two waters pumps (Water Reagent Manager) and a 20 m Teflon coil (2 mL) in a heating reactor (Pickering Laboratories, Mountain View, CA, USA). The chromatographic separation was carried out using a Zorbax Bonus RP column (150 × 4.6 mm, 3.5 μm particle diameter) (Agilent, Santa Clara, CA, USA) with a Zorbax Bonus RP guard column (12.5 × 4.6 mm, 5 μm particle diameter) at 40 °C. For the separation, two mobile phases were used consisting of an 11 mM heptanesulfonate and 5.5 mM phosphoric acid solution adjusted to pH 7.1 with ammonium hydroxide (A) and 11 mM heptane sulfonate, 16.5 mM phosphoric acid and an 11.5% acetonitrile solution adjusted to pH 7.1 with ammonium hydroxide (B). A gradient elution started with a proportion of 100% A, which was maintained for 8 min, followed by a linear increase to 100% B from minute 8.01 to minute 15, and then held for 4 min. The column was equilibrated under the initial conditions for 5 min prior to the next run. The mobile phase flow was 0.8 mL min^−1^ and the injection volume was 10 μL. After separation, the toxins were derivatized in the post-column reaction system (85 °C) with a solution of 100 mM phosphoric acid, 5 mM periodic acid solution adjusted to pH 7.8 with 5 M sodium hydroxide, and, at the end of the reaction coil, mixed with a solution of 0.75 M nitric acid. The flow rate of both solutions was 0.4 mL min ^−1^. Finally, the fluorescent derivatives of the toxins were detected with an FL detector set to 330/390 nm excitation/emission wavelengths. PST concentration in the samples was quantified by comparing the obtained response with that of corresponding reference materials (from IMB-NRC, Ottawa, ON, Canada). The detection limits of the technique were estimated for a signal/noise ratio of 3. Finally, to determine the total toxicity for each sample, toxic equivalency factors were used for calculations and the results were expressed as μg STX diHCL eq 100 g^−1^.

The precision of the method was assessed with repeatability in intra-day (>97%) and inter-day (>95%). The standard solution was analysed in three replicates within 24 h to evaluate the intra-day precision. The inter-day precision was performed by analysis of three replicates for three consecutive days. The retention time was the same for all solutions, showing an oscillating minimum of ≈1.5 min, which was confirmed with toxin-specific standards as well as with the PST standard mix [50].

### 4.5. Statistical Analysis and Graphic Representations

An analysis of variance (ANOVA) was applied to evaluate the effect of weight and technique of analysis (MBA and HPLC) on toxicities. The variations in individual toxicity were evaluated using treatments (foot washed, foot not washed and visceral mass) and whole individual weight, a model selection table based on the Akaike information criteria [70] (AIC) was elaborated showing the number of estimated parameters for each model (K), the delta AIC between comparative models, the Akaike weights, also termed “model probabilities” and the cumulative Akaike weights [71].

All analyses and graphical representations were performed in the R programming environment [72] using the “car” [73], “lmtest” [74] and “AICcmodavg” [75] packages.

## Figures and Tables

**Table 1:** Linear models and generalized linear models (GLMs) assessed to study the individual toxicity variability in edible tissues between treatments and the individual weight of *C. concholepas*. K: number of estimated parameters for each model; AIC: Akaike Information Criteria; Delta AIC: AIC difference between the AIC values ordered from lowest to highest; AIC Wt: Akaike weights; Cum. Wt: cumulative Akaike weights; LL: log-likelihood of each model. * The most informative model.

Model/Hypotheses	K	AIC	Delta AIC	AIC Wt	Cum. Wt	LL
Mouse bioassay						
GLM Gamma: Toxicity = Treatment	4	554.93 *	0	0.63	0.63	−273.21
GLM Gamma: Toxicity = Individual weight + Treatment	5	555.99	1.06	0.37	1	−272.61
GLM negative binomial: Toxicity = Treatment	4	578.28	23.35	0	1	−284.89
GLM negative binomial: Toxicity = Individual weight + Treatment	5	579.59	24.65	0	1	−284.41
Linear model: Toxicity = Individual weight + Treatment	7	784.93	229.99	0	1	−384.73
Linear model: Toxicity = Treatment	3	858.73	303.8	0	1	−426.22
GLM poison: Toxicity = Individual weight + Treatment	4	1654.87	1099.93	0	1	−823.18
HPLC-FLD PCOX						
GLM negative binomial: Toxicity = Individual weight + Treatment	5	727.15 *	0	0.93	0.93	−358.19
GLM Gamma: Toxicity = Individual weight + Treatment	5	732.83	5.67	0.05	0.98	−361.03
GLM Gamma: Toxicity = Treatment	4	736.30	9.15	0.01	0.99	−363.90
GLM negative binomial: Toxicity = Treatment	4	736.62	9.46	0.01	1	−364.06
Linear model: Toxicity = Individual weight + Treatment	7	815.59	88.44	0	1	−400.06
Linear model: Toxicity = Treatment	4	832.54	105.39	0	1	−412.02
GLM poison: Toxicity = Individual weight + Treatment	3	Inf	Inf	0	1	−449.77
GLM poison: Toxicity = Treatment	4	Inf	Inf	0	1	Inf

**Table 2:** Deviance analysis of negative binomial model applied to study toxicity from the bioassay results using variation sources: (i) treatments and (ii) individual weight, to explain the variability of individual toxicity of *C. concholepas*. Findings include linear hypotheses of a posteriori analysis to study the levels of the treatments factor.

	**Degree of Freedom**	**Deviance**	**Residual Degree of Freedom**	**Residual Deviance**	**Pr(>Chi)**
NULL			83	226.233	
Individual weight	1	0.13	82	226.103	0.7185
Treatment	2	144.35	80	81.757	<2 × 10^−16^
Linear hypotheses:					
Treatment factor level		Estimate	Std. error	z value	Pr(>|z|)
Brushed foot − no brushed foot = 0		4.2274	0.3405	−12.415	<1 × 10^−8^
Visceral mas − no brushed foot = 0		1.7131	0.2883	−5.942	<1 × 10^−8^
Visceral mass − brushed foot = 0		2.5142	0.344	7.31	<1 × 10^−8^

**Table 3:** Deviance analysis of the negative binomial model applied to study toxicity from the HPLC-FLD PCOX results using variation sources: (i) treatment and (ii) individual weight to explain the variability of individual toxicity in edible and nonedible tissues of *C. concholepas*. The findings include linear hypotheses of a posteriori analysis to study the levels of the treatments factor.

	**Degree of Freedom**	**Deviance**	**Residual Degree of Freedom**	**Residual Deviance**	**Pr(>Chi)**
NULL			83	245.056	
Individual weight	1	3.73	82	241.326	0.05345
Treatment	2	151.98	80	89.349	<2 × 10^−16^
Linear hypotheses:					
Treatment factor level		Estimate	Std. error	z value	Pr(>|z|)
Brushed foot − no brushed foot = 0		−2.1705	0.1879	−11.553	<0.001
Visceral mass − no brushed foot = 0		−1.6108	0.1859	−8.665	<0.001
Visceral mass − brushed foot = 0		0.5597	0.1907	2.936	0.00933

## Data Availability

Not applicable.
